# Anti-Inflammatory Activity of Compounds Derived from *Vitex rotundifolia*

**DOI:** 10.3390/metabo13020249

**Published:** 2023-02-09

**Authors:** DucDat Le, Sanghee Han, Kyung Hyun Min, Mina Lee

**Affiliations:** 1 College of Pharmacy and Research Institute of Life and Pharmaceutical Sciences, Sunchon National University, 255 Jungangno, Suncheon 57922, Republic of Korea; 2 School of Pharmacy and Institute of New Drug Development, Jeonbuk National University, Jeonju 54896, Republic of Korea

**Keywords:** *Vitex rotundifolia* L. f., vitexrotundifoli A, antioxidant, NO production, IL-8 production, molecular docking

## Abstract

The objective of this study is to describe the separation and identification of one new phenolic and 19 known compounds from *Vitex rotundifolia*. Their structures were determined based on spectroscopic (NMR, CD, and MS) data analysis or Mosher’s method, and were compared with those reported in the literature. These isolates were then evaluated for their anti-inflammatory and antioxidant activities based on the inhibition of nitric oxide (NO) and interleukin (IL)-8 production in lipopolysaccharide (LPS)-stimulated cells (RAW264.7 and HT-29) and 2,2-diphenyl-1-picrylhydrazyl (DPPH) radical scavenging abilities, respectively. In the NO assay, compounds **12**–**14** showed strong inhibition with compounds **10** and **15** displaying significant inhibition. In the IL-8 assay, compounds **8**, **9**, **13**, **14**, **19**, and **20** exhibited potential to inhibit IL-8 production and other compounds displayed moderate inhibition. An in silico docking approach also revealed strong binding affinities for protein–ligand complexes of these active compounds against IL-8 production. The docking results were correlated with the experimental data of the IL-8 assay. Thus, these active compounds should be considered as candidates for further in vivo studies. This study implies the potential of new and active chemicals isolated from *V*. *rotundifolia* and provides evidence to support the development of active fractions and constituents into functional products targeting inflammatory diseases the future.

## 1. Introduction

Inflammatory bowel disease (IBD) is a chronic disease that affects the intestinal mucosa [1]. Specifically, IBD is partly caused by an unbalanced or inappropriate immune response, which has been theorized to be in part directed against gut microbiota [2]. When the immune system is activated, cells are engaged in immune responses by secreting cytokines and chemokines, which set off a chain of subsequent events. As a result, these cytokines are increasingly recognized as crucial players in the development of IBD [3]. They are considered to result from dysregulated expression of molecules engaged in anti-inflammatory processes. These cytokines also show potential as targets for treatment.

Medicinal parts from Genus *Vitex* have been utilized in the management of rheumatoid arthritis [4], and for anti-inflammatory [5] or antioxidant [6] purposes. Among Genus *Vitex*, *V*. *rotundifolia* is a beach plant distributed along the coastal line ranging from Mediterranean regions to Central Asia [7]. Previous studies have reported that this plant contains a variety of chemical derivatives including caffeoylquinic acids, lignans, essential oils, flavonoids, terpenoids, and iridoids with its active constituents showing many biological activities such as antioxidative [8], anticancer [9], and anti-inflammatory [10] activities. In traditional medicine, this plant is used to treat inflammation, rheumatic pain, migraine, and infections [11]. Our previous study [8] revealed that total extracts and fractions of *V*. *rotundifolia* possess potent antioxidant and anti-inflammatory capacities. Ongoing our efforts to find the active constituents from natural sources for the prevention and treatment of inflammation and antioxidant utilization, the objective of this study was to determine its active constituents through biological evaluation using both in vitro and in silico approaches.

## 2. Materials and Methods

### 2.1. Plant Materials

The twigs and leaves of *V*. *rotundifolia* were collected at Goheung (SCNUP 26_SBG-G-1 and -2) in 2021, Korea, and were authorized by Prof. Mina Lee (College of Pharmacy, Sunchon National University). Two voucher specimens were deposited in the Pharmacognosy Lab. (College of Pharmacy, Sunchon National University) in Korea.

### 2.2. Extraction and Separation

Based on our primary research [8], *V*. *rotundifolia* dried leaves (4.68 kg) were extracted with 80% EtOH by sonification for 90 min three times. The soluble residue was concentrated under vacuo to obtain 1.30 kg of total extract. This crude extract was then suspended in water and partitioned with *n*-hexane (H), methylene chloride (MC), EtOAc, and *n*-butanol (B) to obtain H (147.8 g), MC (37.3 g), E (148.7 g), and B (393 g) fractions, and water residue (450 g), respectively. Subsequently, the MC fraction was subjected to MPLC using a biotage C_18_ (120 g) column, eluting with a mobile phase of water (containing 0.1% formic acid, A) and acetonitrile (B) as a gradient solvent system from 0 min (10%, B) to 40 min (70%, B) to obtain 11 fractions (MC1-MC11). Sub-fraction MC4 was subjected to prep-HPLC using a Triart C_18_ (10 × 250 mm, 5 μm) column, eluting with a mobile phase of water (containing 0.3% formic acid, A) and acetonitrile (B) as a gradient solvent system from 0 min (16%, B) to 70 min (25%, B); a wavelength of 254 nm was used to obtain compound **3** (*t*_R_ 30 min). The E-fraction was chromatographed onto silica gel column chromatography and was eluted with a gradient solvent system of methanol in MC from 10 to 100% buffered with 0.01% water to obtain nine fractions (E1-E9). Fraction E1 was subjected to prep-HPLC using a Triart C_18_ (10 × 250 mm, 5 μm) column, eluting with a mobile phase of water (0.3% formic acid, A) and acetonitrile (B) as a gradient solvent system from 0 min (17%, B) to 55 min (90%, B); a wavelength of 254 nm was used to obtain compound **6** (*t*_R_ 17.3 min). Sub-fraction E2 was subjected to ODS open column (5 × 24 cm) chromatography, which was eluted with a gradient solvent system of acetonitrile in water from 17 to 50% (1:5–1:1) to obtain 14 sub-fractions (E2A-E2S). Compound **4** (*t*_R_ 48.4 min) was isolated from the E2C sub-fraction by prep-HPLC using a Triart C_18_ (10 × 250 mm, 5 μm) column, eluting with a mobile phase of water (containing 0.3% formic acid, A) and acetonitrile (B) by an isocratic elution of 0 to 80 min (8%, B). The sub-fraction E2F was subjected to MPLC using a J’sphere ODS-H80 (20 × 250 mm, 4 μm) column, eluting with a mobile phase of water (containing 0.1% formic acid, A) and acetonitrile (B) as a gradient solvent system from 0 min (10% B) to 90 min (40% B) to obtain compound **7** (*t*_R_ 35.2 min), which was further purified on a prep-HPLC using a Triart C_18_ (10 × 250 mm, 5 μm) column eluting with a mobile phase of water (containing 0.1% formic acid, A) and acetonitrile (B) by an gradient elution of 0 min (19%, B) to 80 min (50%, B) to yield compound **9** (*t*_R_ 25.3 min) and compound **12** (*t*_R_ 20.1 min). Fraction E3 was subjected to ODS open column (5 × 24 cm) chromatography, which was eluted with a gradient solvent system of acetonitrile in water from 20 to 33% (1:4–1:2), and was further purified by prep-HPLC using a Triart C_18_ column (10 × 250 mm, 5 μm, YMC, Tokyo, Japan). Detected was performed at a wavelength of 254 nm; flow rate was 3.0 mL/min); and elution was performed with a mobile phase of water (containing 0.3% formic acid, A) and acetonitrile (B) as a gradient solvent system from 0 min (15% B) to 80 min (37% B) to obtain compound **1** (*t*_R_ 43.8 min). Fraction E4 was subjected to ODS open column (8 × 25 cm) chromatography, eluting with a gradient solvent system of acetonitrile in water from 20 to 33% (1:4–1:2) to obtain 26 sub-fractions (E4A-E4Z). The sub-fraction E4F was subjected to MPLC using a J’sphere ODS-H80 (20 × 250 mm, 5 μm) column, eluting with a mobile phase of water (containing 0.1% formic acid, A) and acetonitrile (B) as a gradient solvent system from 0 min (25% B) to 90 min (35% B); a wavelength of 254 nm was used to obtain compound **8** (*t*_R_ 31 min). Compounds **2** and **5** were described in our previous study [8]. Compounds **13** (*t*_R_ 29.7 min) and **14** (*t*_R_ 39.3 min) were isolated from the water residue fraction by YMC-HPLC using a Triart C_18_ (20 × 250 mm, 5 μm) column, using a gradient solvent system from 0 min (13% B) to 65 min (40% B). Similarly, the twig extract (36.68 g) was extracted with 80% EtOH by sonification for 90 min three times. The crude extract was then suspended in water and was successfully partitioned into H (1.0 g), MC (1.53 g), E (9.88 g), and B (9.79 g) fractions, and water residue (15.33 g). Subsequently, the twig H fraction was subjected to a prep-HPLC using a Triart C_18_ (10 × 250 mm, 5 μm) column, eluting with a mobile phase of water (containing 0.1% formic acid, A) and acetonitrile (B) as a gradient solvent system from 0 min (40%, B) to 45 min (90%, B); a wavelength of 254 nm was used to obtain compound **10** (*t*_R_ 49.5 min) and compound **11** (*t*_R_ 44.9 min). The MC fraction was subjected to prep-HPLC using a Triart C_18_ (10 × 250 mm, 5 μm) column, eluting with a mobile phase of water (containing 0.1% formic acid, A) and acetonitrile (B) as a gradient solvent system from 0 min (20%, B) to 55 min (90%, B); a wavelength of 254 nm was used to obtain compound **19** (*t*_R_ 73.7 min) and compound **20** (*t*_R_ 71.5 min). The EA fraction was chromatographed onto silica gel column chromatography, which was eluted with a gradient solvent system of methanol in MC 2 to 100% buffered with 0.01% water to obtain 28 fractions (E1-E28). The E8 sub-fraction was subjected to prep-HPLC using a Triart C_18_ (10 × 250 mm, 5 μm) column, eluting with a mobile phase of water (containing 0.1% formic acid, A) and acetonitrile (B) as a gradient solvent system from 0 min (20%, B) to 55 min (70%, B); a wavelength of 254 nm was used to obtain compound **16** (*t*_R_ 24.8 min). The B fraction was chromatographed onto silica gel column chromatography, which was eluted with a gradient solvent system of methanol in MC from 2 to 100% buffered with 0.01% water to obtain eight fractions (B1-B8). Fraction B5 was subjected to Sephadex open column (3 × 37 cm) chromatography, which was eluted using a 50% MeOH in water to obtain 11 fractions (B5A-B5K). Fraction B5A was subjected to MPLC using a J’sphere ODS-H_80_ (20 × 250 mm, 4 μm) column, eluting with a mobile phase of water (containing 0.1% formic acid, A) and acetonitrile (B) as a gradient solvent system from 0 min (5% B) to 60 min (40% B); a wavelength of 254 nm was used to obtain ten sub-fractions (B5A1-B5A10). Sub-fraction B5A2 was loaded onto the above separation condition using a gradient solvent system from 0 min (2% B) to 50 min (60% B); a wavelength of 254 nm was used to obtain compounds **17** (*t*_R_ 5.1 min) and **18** (*t*_R_ 10 min). Fraction B6 was subjected to ODS open column (5.5 × 25 cm) chromatography, which was eluted with a gradient solvent system of acetonitrile in water (1:10–1:3) to obtain ten sub-fractions (B6A-B6J). Sub-fraction B6E was loaded onto the above separation condition using an isocratic elution of 15% (B) from 0 to 60 min; a wavelength of 254 nm was used to obtain compound **15** (*t*_R_ 54 min).

### 2.3. Modified Mosher’s Method

Compound **1** (1.1 mg for each) was dissolved in anhydrous pyridine (300 µL), followed by the addition of (*S*)-(+)-α-methoxy-α-(trifluoromethyl)phenylacetylchloride (MTPACl) (15 µL) or (*R*)-MTPACl) (15 µL). Subsequently, these reaction mixtures were stirred for 24 h at room temperature. Each mixture ((a) **1** with **1a** or (b) **1** with **1b**) was dried and purified by analytical reversed-phase HPLC to afford **1a** and **1b**. The ∆*δ*_S-R_ values around the stereogenic centers of the MTPA diesters were determined by ^1^H and ^1^H−^1^H COSY NMR spectra.

#### Spectroscopic Data of MTPA Diester Derivatives

(*S*)-MTPA product (**1a**): ^1^H NMR (400 MHz, DMSO-*d*_6_): δ*_H_* 2.04 (1H, m, H-8), 2.74 (1H, d, 19.0, H-3a), 2.96 (1H, m, H-5), 3.38 (overlap, H-3b), 4.41 (2H, t, m, H-9), 4.95 (2H, m, H-7), 5.79 (1H, m, H-4), 7.43 (2H, d, 8.7, H-3’/5’), 8.02 (2H, d, 8.7, H-2’/6’).

(*R*)-MTPA product (**1b**): ^1^H NMR (400 MHz, DMSO-*d*_6_): δ*_H_* 2.07 (1H, m, H-8), 2.57 (1H, d, 19.0, H-3a), 3.11 (1H, m, H-5), 3.40 (overlap, H-3b), 4.46 (2H, t, m, H-9), 4.92 (2H, m, H-7), 5.79 (1H, m, H-4), 7.55 (2H, d, 8.7, H-3’/5’), 8.30 (2H, d, 8.7, H-2’/6’).

### 2.4. Antioxidant Assay

The scavenging DPPH assay was conducted for samples to evaluate their antioxidant capacity. Each well contained 200 μL of sample volume, which was diluted in ethanol to a final concentration of 10 and 100 μM, with 100 μL of DPPH (200 μM) in the ethanol solution. The control was prepared with the same conditions where the amount of sample was replaced by the addition of ethanol. All wells were mixed thoroughly and were incubated at room temperature for 30 min in the shade. When DPPH reacts with an antioxidant sample, the color conversion of deep violet into light yellow was measured at 517 nm with a microplate reader (Epoch, Biotek Instruments, Winooski, VT, USA). Ascorbic acid was used as a positive control for this assay. The antioxidant activity of samples was expressed as the percentage of DPPH reduction between control and treated wells.

### 2.5. NO Assay

#### 2.5.1. Cell Culture and Viability

RAW264.7 cells were purchased from the Korean Cell Line Bank (Seoul, Republic of Korea). They were cultured in Dulbecco′s modified eagle′s medium (DMEM) containing 10% fetal bovine serum (FBS), 100 U/mL penicillin, and 100 μg/mL streptomycin. Cells was grown in a 75T flask at 37 °C in a humidified atmosphere incubator set to 5% CO_2_. RAW264.7 cells (1 × 10^5^ cells/well) were seeded onto 96 well plates and incubated for 24 h before the addition of samples (fractions or pure compounds). LPS (1 μg/mL) stimulation was then applied for 16 h. We assessed the cells using our previous method [8]. RAW264.7 cells were seeded onto 96-well plates and treated with two concentrations of each sample prior to LPS stimulation. Cell viability was assessed by the (3-[4,5-dimethyl-2-thiazolyl]-2,5-diphenyl tetrazolium bromide) (MTT) assay. Cultured cells were incubated with MTT (0.05 mg/mL) at 37 °C for 4 h. The supernatants were then removed and monitored at 570 nm in a microplate reader. The control was prepared per the same conditions without treatment with the sample.

#### 2.5.2. Measurement of NO Production

RAW264.7 cells were incubated with the above samples for 1 h and prior to LPS (1 µg/mL) stimulation. After 16 h, the supernatant was harvested and treated with an equal amount of Griess reagent (equal volumes of 1% (*w*/*v*) sulfanilamide in 5% (*v*/*v*) phosphoric acid and 0.1% (*w*/*v*) naphtylethylene). The cultured supernatant was incubated at room temperature for 10 min before measuring nitrite production using a microplate reader (Biotek Instruments, Inc., Winooski, VT, USA). The control and negative controls were prepared per the same experimental conditions in the presence or absence of LPS stimulation without sample treatment.

### 2.6. IL-8 Assay

#### 2.6.1. Cell Culture and Viability

HT-29 cells were cultured in DMEM medium containing 10% FBS, 100 U/mL penicillin, and 100 μg/mL streptomycin. Cells was grown in a 75T flask at 37 °C in a humidified atmosphere with 5% CO_2_. HT-29 cells (3 × 10^5^ cells/well) was seeded onto 96 well plates, incubated for 2 h before the addition of compounds, and then stimulated with LPS (100 ng/mL) for 12 h. HT-29 cell viability behaved in a similar way to RAW264.7 cell viability in the above conditions.

#### 2.6.2. Measurement of IL-8 Production

The inhibitory effect of compounds (**1**−**20**) on IL-8 production was conducted in LPS-induced HT-29 cells. HT-29 cells were preincubated with compounds (**1**−**20**) for 2 h before the addition of LPS (100 ng/mL). After 12 h, the level of IL-8 in cells was measured using an ELISA kit (BD Biosciences, San Diego, CA, USA), according to manufacturer’s guidelines.

### 2.7. In silico Assay

The 3D structure of IL-8 (PDB ID: 5D14) was obtained from the RCSB protein data bank (https://www.rcsb.org (accessed on 26 October 2022)). Proteins and ligands were prepared using MGL tools 1.5.6. The structures of receptors were processed by removing water, adding polar hydrogen atoms, and Kollman charges. Ligands [3-(*β*-D-glucopyranosyloxymethyl)-2-(4-hydroxy-3-methoxyphenyl)-5-(3-hydroxypropyl)-7-methoxy-(2*R*,3*S*)-dihydrobenzofuran (**8**), chrysosplenol-D (**9**), luteolin-7-glucopyranoside (**13**), luteolin-5-glucopyranoside (**14**), and ursolic acid (**19**)] were downloaded from Pubchem (https://pubchem.ncbi.nlm.nih.gov) with sdf formats, and were transformed into pdbqt formats using the Open Babel program (version 3.1.1). The ligand conformations were performed by adding Gasteiger charges. A grid box of coordinates was referred from a previous study [12]. A total of 1000 runs were conducted under default parameters using Lamarckian genetic algorithm. The protein–ligand docking calculations were performed using Autodock vina 1.1.2 software. Residue–ligand interactions were visualized with the Discovery studio 2021 tool (version V21.1.0.20298) and Pymol program (version 2.5.3).

### 2.8. Statistical Analysis

Data were represented as the mean ± standard deviation (SD) (*n* = 3) of three replicates. The nonparametric one-way ANOVA followed by Dunnett’s multiple comparison test was performed using Graphpad Prism (version 8.0.1) software (La Jolla, CA, USA). * *p* < 0.05, ** *p* < 0.01, compared to controls, was accepted as statistically significant.

## 3. Results

### 3.1. Separation and Identification of Chemical Constituents

**Figure 1:**
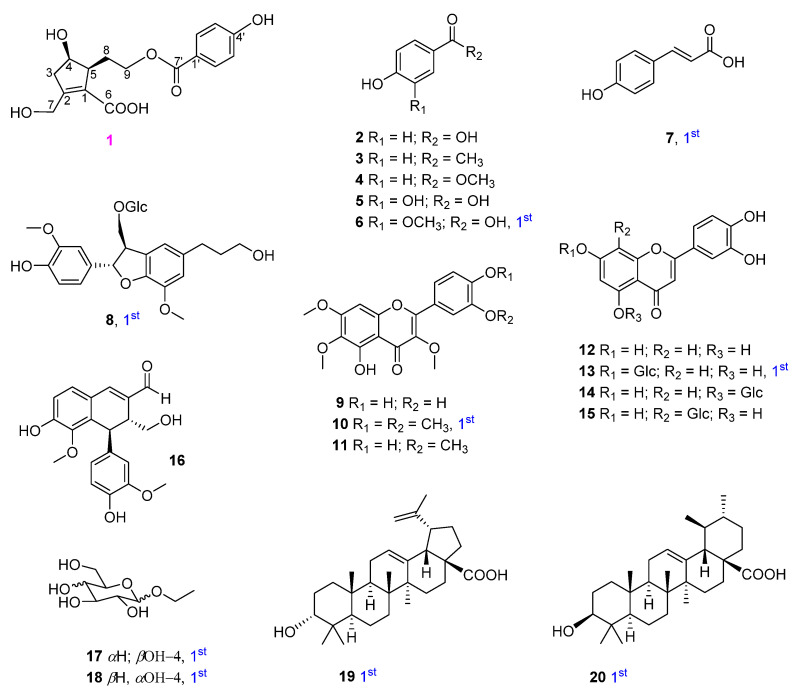
Structures of isolated compounds **1**−**20** from *V*. *rotundifolia*.

Using multiple chromatographic techniques, 20 compounds (one new phenolic, six monophenyl esters, two lignans, seven flavones, two glucosides, and two triterpenoids) were isolated; their structures were then elucidated using chemical reactions and analysis of their spectroscopic data was performed. Their structures are depicted in Figure 1.

### 3.2. Structural Determination of Compounds (**1**−**20**)

**Figure 2:**
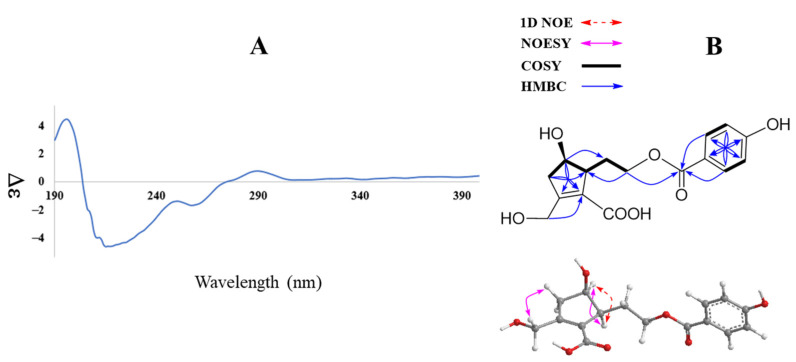
CD spectrum (**A**) and key HMBC, COSY, 1D NOE, and NOESY (**B**) correlations of compound **1**.

Compound **1** was obtained as an amorphous powder. The infrared (IR) spectrum of **1** showed absorption of the following functional groups: OH (3374 cm^−1^), carbonyl (1696 cm^−1^), and olefinic (1600 cm^−1^). Its high mass spectrum showed a deprotonated ion [M—H]^–^ peak at m/z 321.0984 (calcd. for C_16_H_17_O_7_, 321.0974). The ^1^H NMR spectrum of **1** displayed characteristic signals of an aromatic AABB spin system at δ_H_ 7.81 (2H, d, J = 8.7 Hz, H-2’/6’) and 6.84 (2H, d, J = 8.7 Hz, H-3’/5’); four methylene groups at δ_H_ 4.40 (H-7), 4.25 and 4.18 (H-9), 2.84 and 2.42 (H-3), 1.94 and 1.62 (H-8); and two methines at δ_H_ 4.01 (H-4) and 2.82 (H-5) (Table 1). The ^13^C NMR spectrum of **1** showed 14 carbons including two carbonyl signals at δ_C_ 166.6 (C-6) and 165.6 (C-7’); six aromatic carbons at δ_C_ 161.9 (C-4’), 131.4 (C-2’/6’), 120.5 (C-1’), and 115.3 (C-3’/5’); together with two olefinic carbons at δ_C_ 157.1 (C-2) and 128.2 (C-1); three oxygenated carbons at δ_C_ 72.4 (C-4), 62.8 (C-9), and 58.9 (C-7); a methine at δ_C_ 53.1 (C-5); and two methylene signals at δ_C_ 43.4 (C-3) and 30.2 (C-8) (Table 1) (Dept spectrum, Figure S6, Supplementary Material). Protons and carbons assignments were further confirmed by HMQC and Dept spectra. The ^1^H−^1^H COSY spectrum showed a sequence of correlations of H-3/ H-4/ H-5 together with HMBC correlations from H-3 (δ_H_ 2.42 and 2.84)/ H-4 (δ_H_ 4.01)/ H-5 (δ_H_ 2.82) to C-1 (δ_C_ 128.2)/ C-2 (157.1), which established the cyclopentene moiety. In addition, HMBC correlations from the methylene group at δ_H_ 4.40 to C-1 (δ_C_ 128.2)/ C-2 (δ_C_ 157.1)/ C-3 (δ_C_ 43.4) suggested a 7-CHOH combination. ^1^H−^1^H COSY correlations of H-5/H-8/H-9 were also observed. HMBC correlations of H-8 (δ_H_ 1.62 and 1.94) to C-1/C-4/C-5 indicated C-5, C-8, C-9 assignment. The HMBC spectrum exhibited correlations of H-9/H-2’/H-6’ to C-7’, which confirmed 4-hydroxybenzoyl moiety attachment through the C-9 position (Figure 2B).

Further COSY and HMBC correlations are shown in Figure 2B. The relative configuration of **1** was established by analysis of NOESY correlations and the 1D NOE spectrum. Indeed, the NOESY spectrum showed a correlation of H-4 (δ_H_ 4.01) to H-5 (δ_H_ 2.82) and those of H_2_-3 (δ_H_ 2.42 and 2.84) to H-7 (δ_H_ 4.40). Furthermore, selective 1D NOE irradiation of H-4 showed a significant enhancement at H-5. The above observation indicated a sym-form of H-4 and H-5.

**Figure 3:**
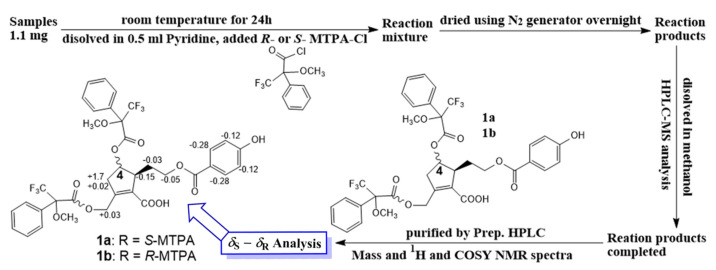
Modified Mosher’s method.

In addition, the experimental CD spectrum was analyzed to determine the absolute configuration of chiral centers of compound **1**. The CD spectrum (Figure 2A) of **1** showed negative Cotton effects at about 220 and 260 nm [13], indicating 4R and 5S configurations, respectively. Regarding the overall consideration, the OH-4 configuration was determined by a modified Mosher’s method. Δδ values of the (S)- and (R)-MTPA diesters (**1a** and **1b**) (Figure 3) confirmed the C-4R orientation. Based on the collected evidence, the absolute configuration of **1** was judged to have C-4R and C-5S. Therefore, the new compound **1** was elucidated as an isomer of a reported compound [13]. It was named vitexrotundifoli A.

In addition, 19 other compounds were isolated and their structures were identified as 4-hydroxybenzoic acid (**2**) [14,15], 4′-hydroxyacetophenone (**3**) [16], 4-hydroxybenzoic methyl ester (**4**) [15], protocatechuic acid (**5**) [17], vanillic acid (**6**) [18], trans-4-hydroxycinnamic acid (**7**) [19], 3-(β-D-glucopyranosyloxymethyl)-2-(4-hydroxy-3-methoxyphenyl)-5-(3-hydroxypropyl)-7-methoxy-(2R,3S)-dihydrobenzofuran (**8**) [20], chrysosplenol D (**9**) [21], artemetin (**10**) and casticin (**11**) [22], luteolin (**12**) and luteolin-7-glucopyranoside (**13**) [23], luteolin-5-glucopyranoside (**14**) [24], orientin (**15**) [25], vitedoin A (**16**) [6], ethyl α-D-glactopyranoside (**17**) [26], ethyl β-D-glucopyranoside (**18**) [27], betulinic acid (**19**) [28], and ursolic acid (**20**) [29] by comparing their spectroscopic data to those reported in the literature. Among them, compounds **6**−**8**, **10**, **13**, and **17**−**20** were isolated from *V*. *rotundifolia* for the first time.

### 3.3. Biological Activities of Isolated Compounds

**Figure 4:**
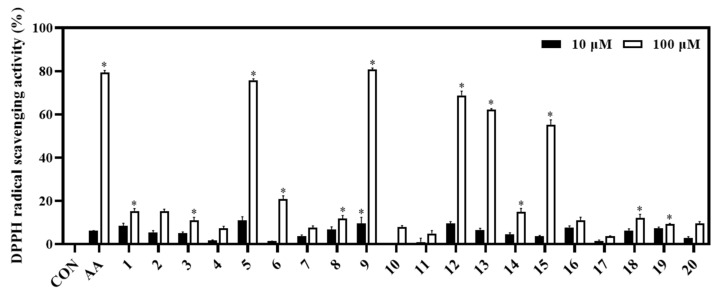
Antioxidant effect of compounds (**1**−**20**). The DPPH assay was performed in triplicate. The data are represented as mean ± SD. * *p* < 0.05, compared to control (CON).

The biological capacities of isolates (**1**−**20**) were evaluated based on their antioxidant and anti-inflammatory activities through their inhibitory abilities on DPPH radical scavenging, NO production, and IL-8 production, respectively. Among them, compounds **5** and **9** showed potent DPPH radical scavenging abilities (inhibition at 75.84% and 80.83%, respectively), relative to the positive control (ascorbic acid, **AA;** inhibition at 79.31% at a concentration of 100 μM). Whereas compounds **12**, **13**, and **15** displayed significant antioxidant effects with DPPH radical scavenging abilities of 68.80%, 63.50%, and 51.42%, respectively (Figure 4).

**Figure 5:**
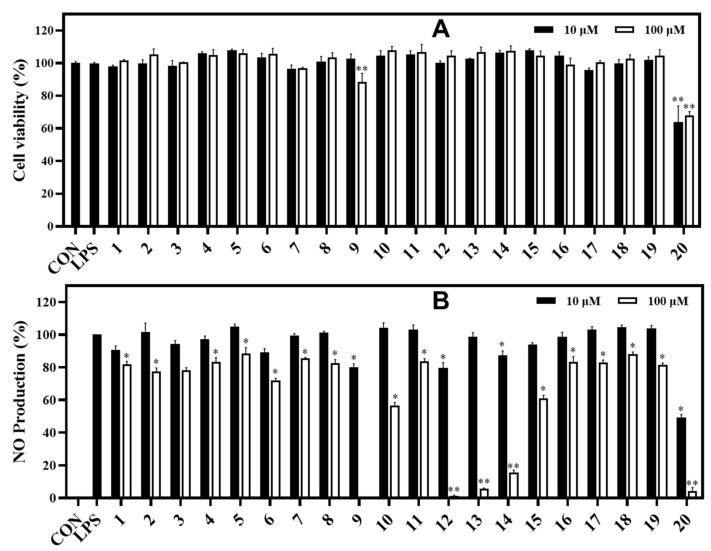
Cytotoxic effects (**A**) and NO production inhibitory effects (**B**) of isolated compounds (**1**−**20** at 10 and 100 μM) in LPS-stimulated RAW264.7 cells. RAW264.7 cells were treated with compounds **1**−**20** (10 and 100 µM) for 1 h and stimulated with LPS (1 μg/mL) for 16 h. (**A**) The viability of cells was determined using an MTT assay. (**B**) The level of NO production in serum-free culture medium was measured. Both experiments were performed in triplicate. The data are represented as mean ± SD. *p < 0.05, **p < 0.01, compared to the LPS-treated group.

In the NO assay, compounds **9** and **12**−**14** exhibited strong inhibition against NO production in LPS-stimulated RAW264.7 cells, with inhibitory rates of 99.71%, 98.84%, 94.46%, and 84.55%, respectively, at 100 μM. Compounds **10** and **15** showed significant inhibition (43.30% and 40.51%, respectively) under the same test conditions. In contrast, the active compounds **9**, **10**, and **12**−**15** did not exhibit significant cytotoxicity, which was verified by the MTT assay (Figure 5). Compound **20** displayed a strong inhibition against NO production with rate of 95.62% at the tested concentration of 100 μM. However, compound **20** exhibited a cytotoxic effect on RAW264.7 cells based on the MTT assay. After treatment with the other compounds, the NO production was weakly reduced in LPS-stimulated cells, which did not show any proliferation (Figure 5).

**Figure 6:**
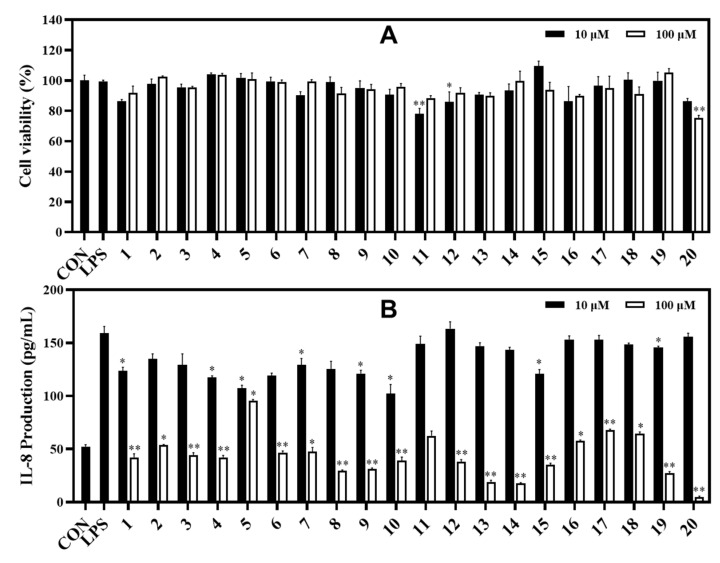
Cytotoxic effects (**A**) and IL-8 production inhibitory effects (**B**) of compounds in LPS-induced HT-29 cells. HT-29 cells were treated with compounds **1**−**20** (10 and 100 µM) for 2 h and stimulated with LPS (100 ng/mL) for 12 h. (**A**) The viability of cells was determined using an MTT assay. (**B**) The level of IL-8 in the culture media was measured with an ELISA kit. The values are expressed as mean ± standard deviation of three individual experiments. * *p* < 0.05, ** *p* < 0.01, compared to the LPS-treated group.

In the IL-8 assay, compounds **13** and **14** potently reduced LPS-induced IL-8 production in HT-29 cells with inhibition rates of 81.03% and 82.39%, respectively, compared to the control at the tested concentration of 100 μM. Under the same test conditions, compounds **8**, **9**, and **19** showed strong inhibition against IL-8 production with inhibition rates of 70.39%, 68.58%, and 72.59%, respectively. Compounds **1**−**4**, **6**, **7**, **10**−**12**, and **15**−**18** exhibited significant inhibition against IL-8 production at rates ranging from 64.98% to 32.35%. Compound **20** strongly inhibited IL-8 production at a concentration of 100 μM. However, compounds **11** and **20** also showed some toxic effects when measuring cell viability (78.07% and 75.29%, respectively) of HT-29 cells. Whereas other compounds displayed cell viability effects ranging from 86.43% to 109.54% at both tested concentrations of 10 and 100 μM using the MTT assay (Figure 6).

### 3.4. In silico Approach Analysis

**Figure 7:**
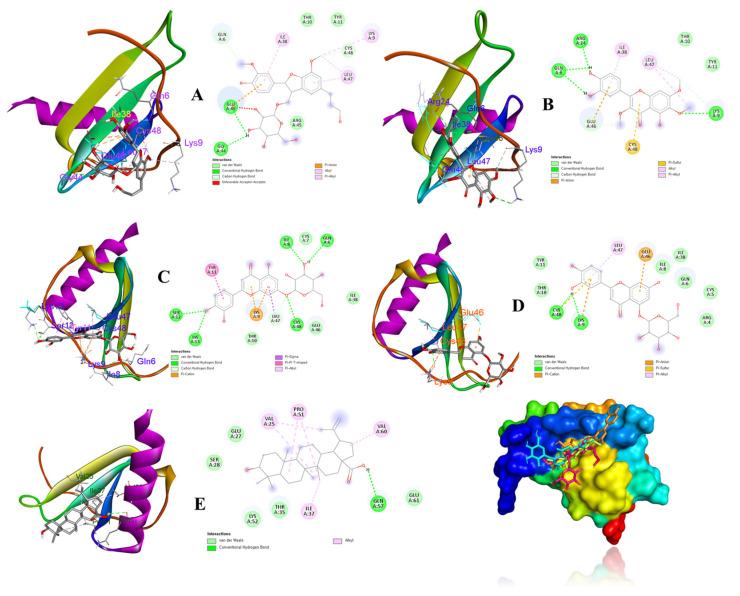
Binding poses and interactions between binding sites of the IL-8 receptor with respect to ligands (compounds **8** (**A**), **9** (**B**), **13** (**C**), **14** (**D**), and **19** (**E**)).

According to above results, compounds **8**, **9**, **13**, **14**, and **19** were considered as ligands to evaluate their binding affinities for the target IL-8 protein (PDB ID: 5D14) using a molecular docking study. Docking results and the interactions of the above compounds isolated from *V*. *rotundifolia* in the ligand–receptor complexes surrounded by the critical amino acids of the IL-8 protein are depicted in Figure 7 The results revealed that ligands (compounds: **13**, −8.3 kcal/mol; **14**, −8.4 kcal/mol) exhibited strong binding affinities for the 5D14 receptor protein. Ligands (compounds: **8**, −7.5 kcal/mol; **9**, −6.6; and **19**, −7.8 kcal/mol) also exhibited energetically favorable binding poses surrounded by active sites with hydrogen bonds and other interactions with active residues of the IL-8 protein (Table 2 and Figure 7).

## 4. Discussion

In purpose of finding active constituents from a herbal medicine, our current study demonstrated the isolation and identification of 20 chemicals, including a novel compound (**1**) and nine compounds firstly isolated from *V*. *rotundifolia*, which were classified into seven known phenolics, two lignans, seven flavones, two glycosides, and two triterpenoids. The structures of the isolates were elucidated by analysis of their spectroscopic and mass data. The structure of the new compound, vitexrotundifoli A, was successfully determined by analysis of its 1D, 2D NMR, and high mass data. Specially, its configuration was roughly established using 1D NOE and 2D NOESY, and then was approved by the modified Mosher’s method together with the CD spectrum. Our chemical investigation showed the diversity of secondary metabolites from this plant.

When evaluating the activities of the isolated compounds, our results highlighted the medicinal values of this plant through the inhibitory effects of the compounds on biological activities such as antioxidant and anti-inflammatory activity. Briefly, compounds **5**, **9**, **12**, and **13** exhibited strong DPPH radical scavenging activity with inhibition rates of 75.84%, 80.83%, 68.80%, and 63.50%, respectively, clarifying the DPPH radical scavenging capacity of their fraction-derived compounds. This observation also revealed that some activated functional groups could enhance the antioxidant activity. Briefly, compounds **2**−**7** are monophenyl esters; however, compound **5** showed the highest antioxidant effect, which was higher than the other compounds (**2**−**4**, **6**, and **7**). Thus, the 5-OH functional group is important for the antioxidant activity. Compounds **9**−**11** had the same flavone backbone. Among them, compound **9** displayed the strongest antioxidant effect. This implies that 4’- and 5’-OCH_3_ functional groups do not favor DPPH radical scavenging activity. Compounds **12**−**15** belong to luteolin derivatives. Among them, compound **14** had a glycosidation at C-5. It possesses the lowest radical scavenging ability, which was lower than compounds **12**, **13**, and **15**. Thus, luteolin derived C-5-O-glc linkage might decrease antioxidant activity. This is the first report on the DPPH radical scavenging activity of ethyl α-D-glactopyranoside.

These isolates also exhibited a significant inhibition of LPS moderation of NO production. At the tested concentration of 100 μM, compound **9** showed the most potential effect with an inhibition rate of NO production at 99.71%, following by compounds **12** (98.84%), **13** (94.46%), and **14** (84.55%) at 100 μM. The biological activity results of these luteolin derivatives also showed good anti-inflammatory effects in previous studies [30,31]. Similar results were also observed for flavone compounds (**9**−**11**), indicating that C-8-glc might not improve the inhibitory effect of NO production in the tested conditions. Furthermore, this is the first report of ethyl *α*-D-glactopyranoside (**17**) modulating LPS-induced NO production in RAW264.7 cells.

In this study, luteolin compounds ((**12**−**15**) showed significant bioactivities in both DPPH and NO assays. This observation is in line with previous reports [32,33,34] for luteolin (3′,4′,5,7-tetrahydroxyflavone) derivatives regarding to their abilities to regulate inflammatory mediators or radical generators in a dose-dependent fashion.

In the IL-8 assay, compounds **13** and **14**, which are luteolin glycosides, strongly inhibited IL-8 production in LPS-stimulated HT-29 cells. Whereas compounds **8**, **9**, and **19** displayed significant induction of IL-8 production with inhibition rates ranging from 68.58% to 72.59%, respectively. The new compound (**1**) and other compounds (**2**−**7**, **10**−**12**, and **15**) exhibited a moderate reduction in IL-8 production at the same tested concentration of 100 μM. A structural activity relationship was deducted from the above results. Compounds (**2**−**7**) are monophenyl esters. Among them, compound **5** showed the weakest activity. Therefore, the 5-OH functional group may reduce the inhibitory effect in LPS-modulated IL-8 production on HT-29 cells in the tested conditions. Interestingly, the anti-inflammatory effect on LPS-induced IL-8 production of compounds **8**, **9, 17**, and **18** was reported for the first time in HT-29 cells in this study.

The active compounds (**8**, **9**, **13**, **14**, and **19**) were docked onto the target IL-8 protein, respectively, to investigate and predict the possible mechanisms of inhibition of the inflammatory cytokine by molecular docking. The docking scores (Table 2) revealed that their binding energies toward the IL-8 receptor were associated with their inhibitory effects on IL-8 production in LPS-stimulated HT-29 cells in the experimental data (Figure 6B). Some evidence supported that compounds **13** and **14** showed strong binding affinities to IL-8 protein, showing hydrogen bonds for Gln6, Ile8, Ser12, Lys13, Cys48, and Lys9, and Cys48, respectively. Additionally, compounds **8** and **9** exhibited hydrogen interactions with Gly44 and Glu46, and Gln6, Lys9, and Arg24 amino acids, respectively. On the other hand, compound **19** displayed hydrogen bonds to Gln57 residues of the IL-8 protein. These active sites of the receptor might be key factors for binding and interactions of the ligand–receptor complexes.

In the NO and IL-8 assays, compound **20** showed strong inhibitory effects for both experiments. However, this compound also displayed anti-proliferation effects on cells (RAW264.7 and HT-29). Therefore, compound **20** may be considered as a good candidate as it has cytotoxic capacity for anticancer and antitumor activities. The cytotoxicity of compound **20** (ursolic acid) was due to its structure of triterpenoids known to show antiproliferation effects on many cancer cell lines, including MDA-MB-231 human breast cancer cells [35] and B164A5 murine melanoma cells [36], in previous studies.

Our research identified the bioactive components with antioxidant and anti-inflammatory properties from plant extracts that were intended to have anti-inflammatory actions [37] to increase their use in ethnopharmacological purposes for treating inflammation [11].

## 5. Conclusions

In conclusion, a phytochemical investigation from herbal medicine, *V*. *rotundifolia*, resulted in isolation and determination of 20 compounds, one new, nine firstly isolated, and ten known compounds, using multiple chromatographic techniques and spectroscopic data. Various spectroscopies (1D/2D-NMR, CD, MS) and chemical reactions were applied to identify the structures of these compounds, which were further compared to those reported in the literature. The results of this study’s activity analysis, which identified components with anti-inflammatory and antioxidant actions through NO, IL-8, and DPPH assays, significantly improved the plant’s traditional usage. Our work, which builds on the scientific underpinnings of our earlier investigation, is focused on the active components of this plant, which has been historically utilized as a medication in relation to its anti-inflammatory effects. The present results further imply that enrichment of fraction-derived active ingredients may result in the creation of functional products targeted at disease prevention and therapy with anti-inflammatory and antioxidant properties.

## Figures and Tables

**Table 1:** Spectroscopic data of compound **1**.

No.	1	
	*δ*_C_	*δ*_H_ (mult., *J* = Hz)
1	128.2	-
2	157.1	-
3	43.4	2.42 (1H, d, 19.0) 2.84 (1H, dd, 5.5, 19.0)
4	72.4	4.01 (1H, d, 6.0)
5	53.1	2.82 (1H, d, 5.5)
6	166.6	-
7	58.9	4.40 (2H, brs)
8	30.2	1.62 (1H, ddd, 4.8, 9.4, 14.2) 1.94 (1H, td, 3.7, 7.1, 14.2)
9	62.8	4.18 (1H, m) 4.25 (1H, m)
1’	120.5	-
2’/6’	131.4	7.81 (2H, d, 8.7)
3’/5’	115.3	6.84 (2H, d, 8.7)
4’	161.9	-
7’	165.6	-

NMR data of compound **1** was recorded in DMSO-*d*_6_.

**Table 2:** Binding energies and interactions of active compounds against IL-8 production docked into active sites of the IL-8 protein.

Compounds	Docking Score (kcal/mol)	Conventional Hydrogen Bond	Other Interactions
8	−7.5	Gly44, Glu46	Gln6, Lys9, Thr10, Tyr11, Arg45, Leu47
9	−6.6	Gln6, Lys9, Arg24	Thr10, Tyr11, Ile38, Glu46, Leu47, Cys48
13	−8.3	Gln6, Ile8, Ser12, Lys13, Cys48	Cys7, Lys9, Thr10, Tyr11, Ile38, Glu46Leu47
14	−8.4	Lys9, Cys48	Arg4, Cys5, Gln6, Ile8, Thr10, Tyr11, Ile38, Glu46, Leu47
19	−7.0	Gln57	Val25, Glu27, Ser28, Thr35, Ile37, Pro51, Lys52, Val60, Glu61

## Data Availability

The data presented in this study are available in article and supplementary material.

## Supplementary Materials

The following supporting information can be downloaded at: https://www.mdpi.com/article/10.3390/metabo13020249/s1. Figure S1: Separation of compounds (**1**−**9** and **12**−**14**) from leaves of *V*. *rotundifolia*. Figure S2: Separation of compounds (**10**, **11**, and **15**−**20**) from leaves of *V*. *rotundifolia*. Figure S3: HR-MS spectrum of compound **1**. Figure S4–S11: Spectroscopic spectra of compound **1**. Figure S12: CD spectrum of compound **1.** Figure S13: Low mass spectrum of MTPA ester product. Figure S14: ^1^H NMR spectra of **1a** and **1b**. Figure S15: COSY spectra of **1a**. Figure S16: COSY spectra of **1b**.
