# Supplementary material for: Anti-Inflammatory Activity of Compounds Derived from Vitex rotundifolia
Source: Metabolites. 2023 Feb 9;13(2):249. doi: 10.3390/metabo13020249 (PMC9962727; doi:10.3390/metabo13020249)
Supplement: Supplementary file 1 [file metabolites-13-00249-s001.zip › metabolites-2165703-supplementary.pdf]

Supplementary material

Anti-inflammatory activity of compounds derived from *Vitex rotundifolia*

DucDat Le[1], Sanghee Han[1], Kyung Hyun Min[2,*] and Mina Lee[1,*]

[1]College of Pharmacy and Research Institute of Life and Pharmaceutical Sciences, Sunchon National Uni-versity, 255 Jungangno, Suncheon 57922, Jeonnam, Korea
[2]School of Pharmacy and Institute of New Drug Development, Jeonbuk National University, Jeonju 54896, Korea

*Corresponding author: Kyung Hyun Min
🖂 E-mail: khmin1492@jbnu.ac.kr(K.H.M); Tel.: +82-63-219-5651; Fax: +82-63-219-5638.

*Corresponding author: Mina Lee
🖂 E-mail: minalee@sunchon.ac.kr/minalee@scnu.ac.kr; Tel.: +82-61-750-3764; Fax: +82-61-750-3708.

Table of Contents

1. General procedures and chemical reagents:......1

2. Extraction and separation of compounds (1–20):......2

Figure S1. Separation of compounds (**1**–**9** and **12**–**14**) from leaves of *V. rotundifolia*......2

Figure S2. Separation of compounds (**10**, **11**, and **15**–**20**) from leaves of *V. rotundifolia*......2

3. Spectroscopic data of new compounds 1:......3

Figure S3. HR-MS spectrum of compound **1**......3

Figure S4. ^{1}H NMR (400 MHz, DMSO-d_6) spectrum of compound **1**......3

Figure S5. ^{13}C NMR (400 MHz, DMSO-d_6) spectrum of compound **1**......4

Figure S6. DEPT spectrum of compound **1**......4

Figure S7. ^{1}H-^{1}H COSY spectrum of compound **1**......5

Figure S8. ^{1}H-^{13}C HMQC spectrum of compound **1**......5

Figure S9. ^{1}H-^{13}C HMBC spectrum of compound **1**......6

Figure S10. ^{1}H-^{1}H NOESY spectrum of compound **1**......6

Figure S11. Selective 1D NOE spectrum of compound **1**......7

Figure S12. CD spectrum of compound **1**......7

Figure S13. Low mass of MTPA diester product......8

Figure S14. ^{1}H NMR spectra of **1a** and **1b**.......8

Figure S15. COSY spectrum of **1a**.......9

Figure S16. COSY spectrum of **1b**......9

1. General procedures and chemical reagents:

The NMR spectra of isolates **1**–**20** were operated on a JEOL spectrometer (JNM-AL 400 MHz, JEOL, Tokyo, Japan). The chemical shifts were detected as δ values (ppm) with TMS as the internal standard (recorded in DMSO-d_6 and CD_3OD). The mass spectra was performed on a Thermo scientific Vanquish UHPLC system (Thermo Fisher Scientific, Sunnyvale, CA, USA) coupled with Shimadzu column and a triple TOF 5600+ mass spectrometer system (Triple TOF MS) (SCIEX Foster City, CA, USA). Column chromatography (CC) was performed on silica gel (Kieselgel 60, 70–230 mesh and 230–400 mesh, Merck, Darmstadt, Germany), thin layer chromatography (TLC) using precoated RP-18 F_{254S} plates (1.15685.0001, Merck) and silica gel 60 F_{254} (1.05554.0001, Merck), visualized by heating for 1.5–2 min after spraying with aqueous 10% H_2SO_4. HPLC was performed using a Waters 2695 with UV 996 Photodiode array detector and YMC Pak ODS-A column (20 × 250 mm, 5 μm particle size, YMC Co., Ltd., Kyoto, Japan). HPLC solvents were purchased from Burdick & Jackson, MI, USA and Fisher Chem Alert Guide, PA, USA. All other chemicals and solvents of analytical grade were purchased and used without purification. Dulbecco's modified Eagles medium (DMEM), fetal bovine serum (FBS), penicillin, and streptomycin were purchased from Hyclone (HyClone, Logan, UT, USA). Bacterial LPS (Escherichia coli 111: B4) was purchased from Sigma-Aldrich (St. Louis, MO, USA). Enzyme-linked immunosorbent assay (ELISA) kits for NO and IL-8 were purchased from BD Biosciences (San Diego, CA, USA). A protein-extraction solution was purchased from IntRon (Sungnam, Korea). Antibodies such as iNOS, COX-2, and *β*-actin were purchased from Santa Cruz Biotechnology (Santa Cruz, CA, USA). Super-Signal chemiluminescent substrate was purchased from Thermo Fisher Scientific (Wal-tham, MA, USA).

2. Extraction and separation of compounds (1–20):

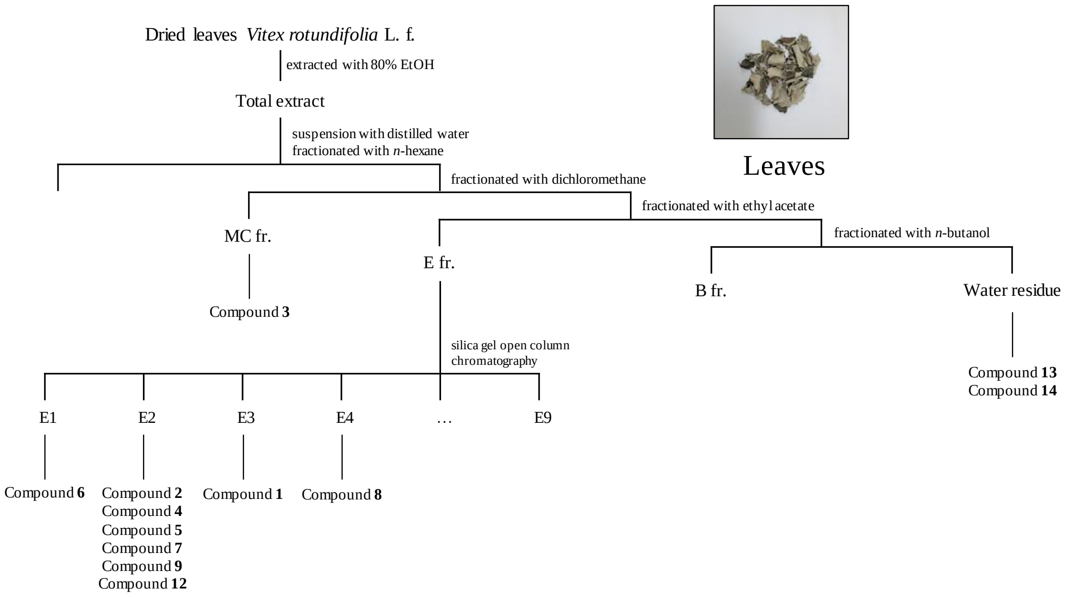

Figure S1. Separation of compounds (**1**–**9** and **12**–**14**) from leaves of *V. rotundifolia*.

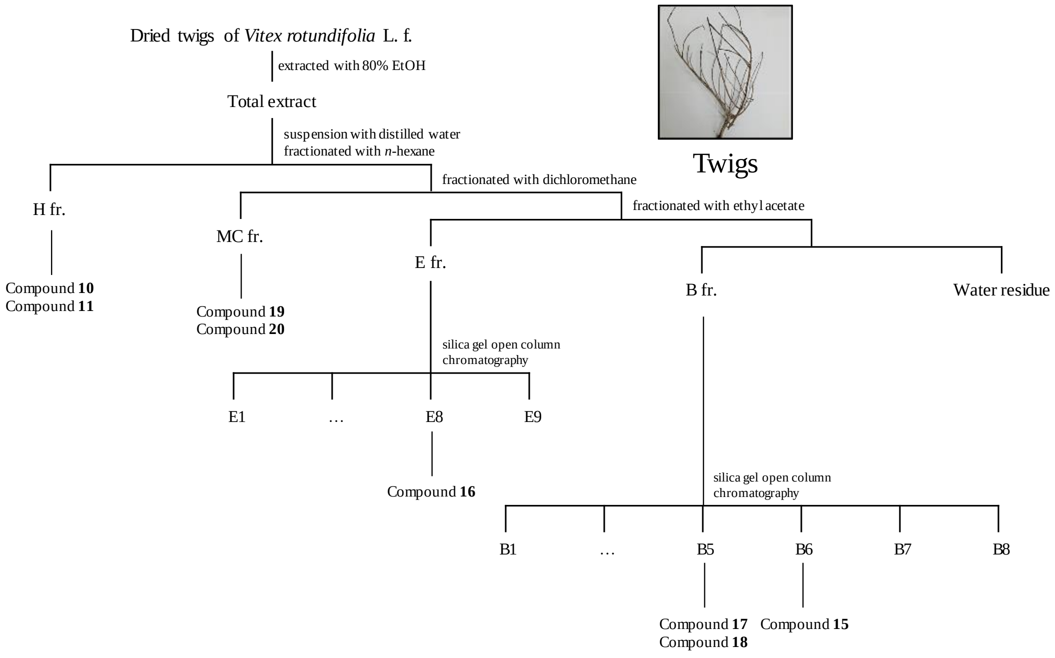

Figure S2. Separation of compounds (**10**, **11**, and **15**–**20**) from twigs of *V. rotundifolia*.

3. Spectroscopic data of new compound (1):

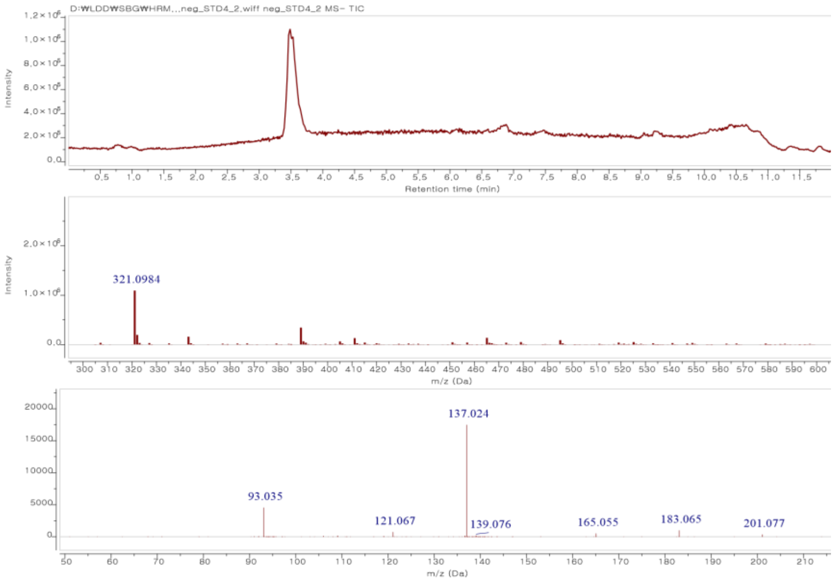

Figure S3. High mass spectrum of compound **1**.

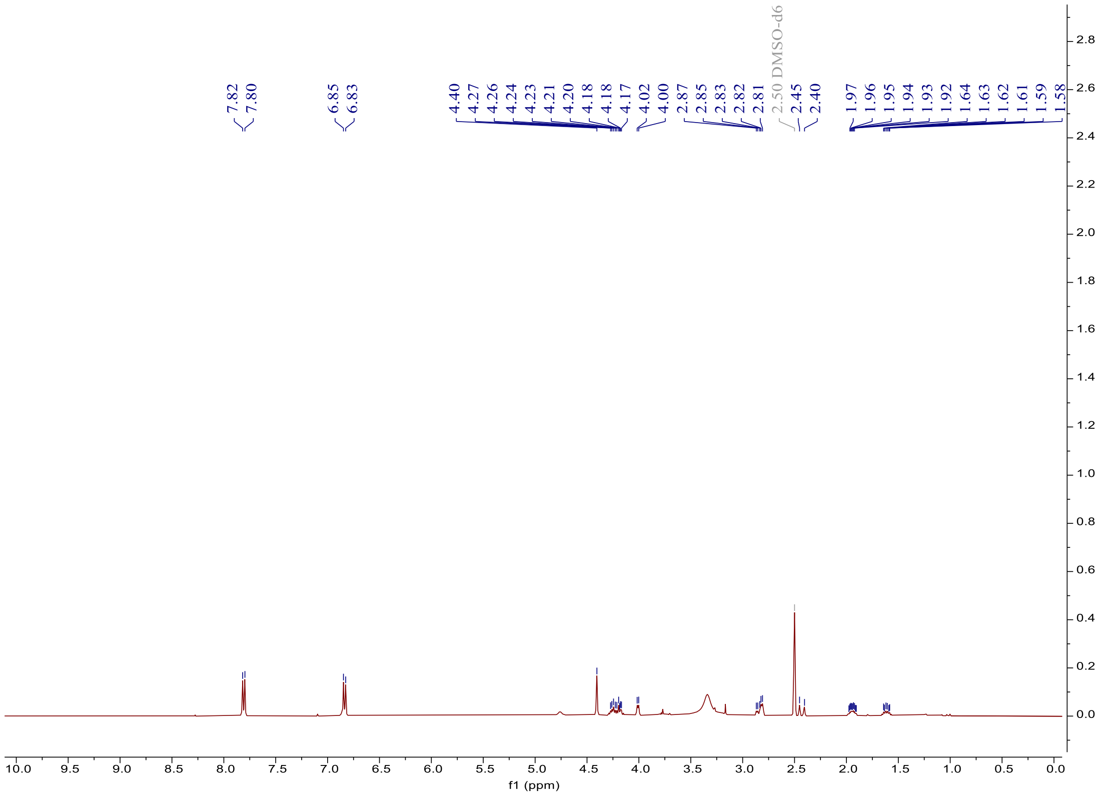

Figure S4. ^{1}H NMR (400 MHz, CD_3OD) spectrum of compound **1**.

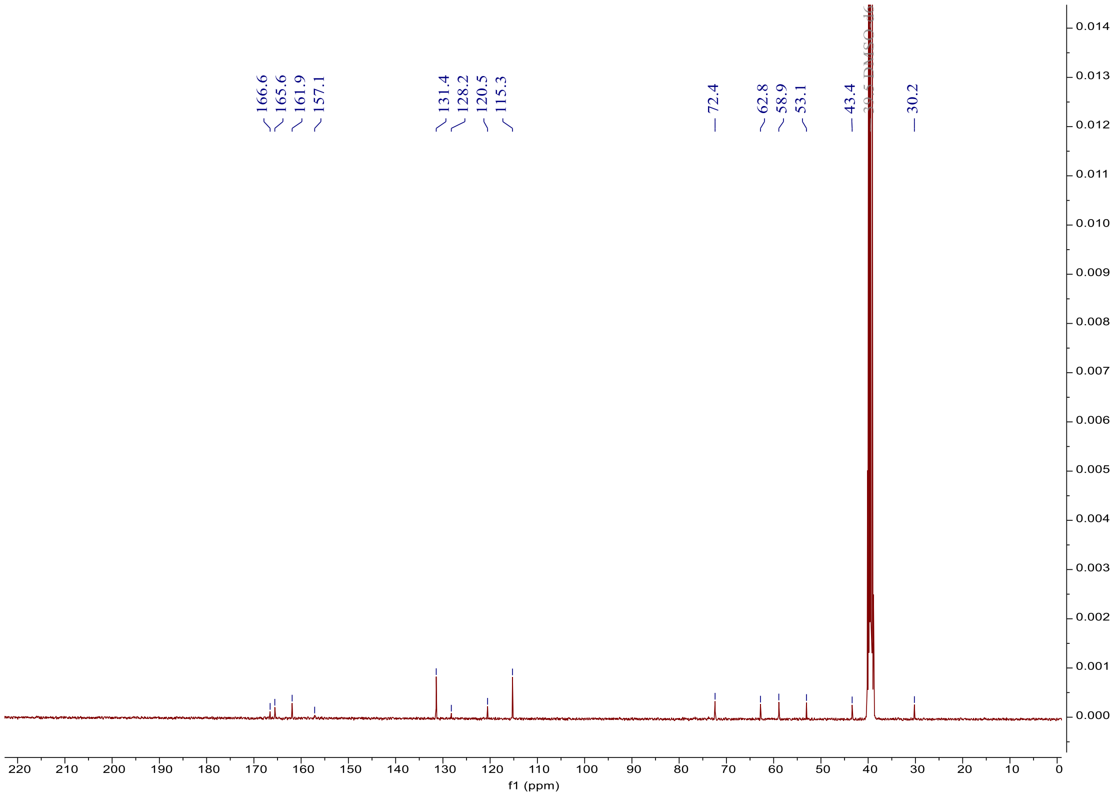

Figure S5. ^{13}C NMR (100 MHz, CD_3OD) spectrum of compound **1**.

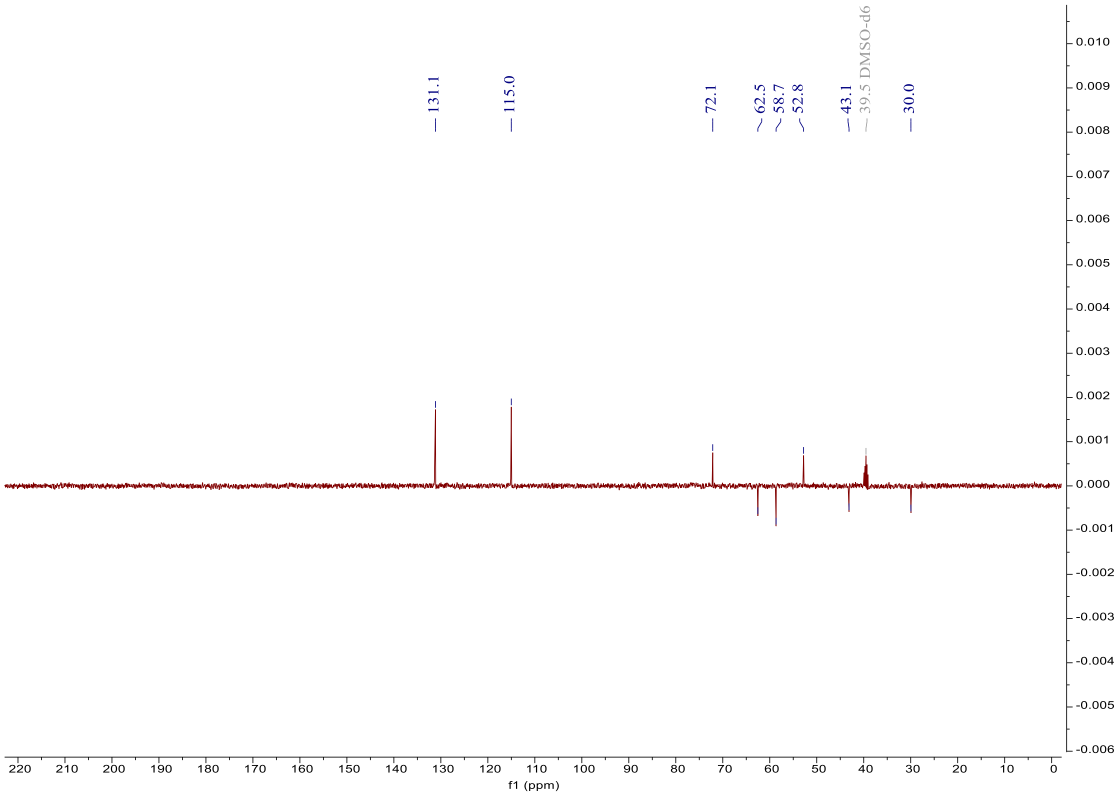

Figure S6. DEPT spectrum of compound **1**.

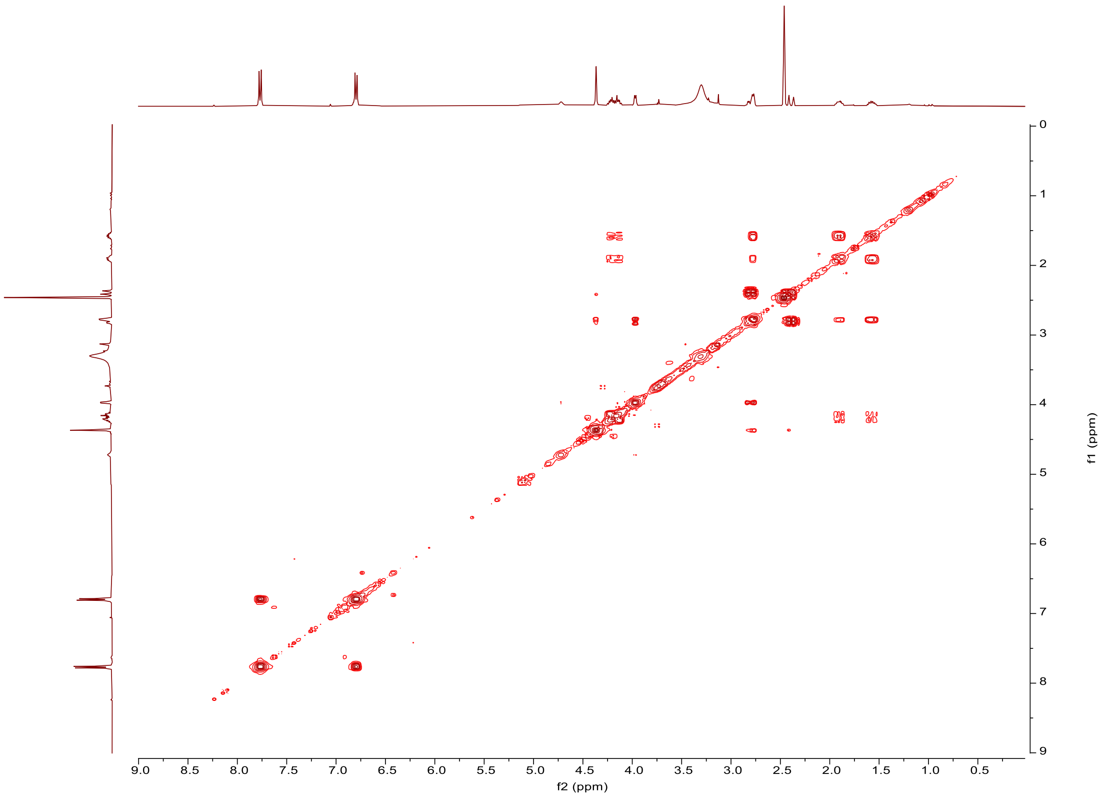

Figure S7. ^{1}H-^{1}H COSY spectrum of compound **1**.

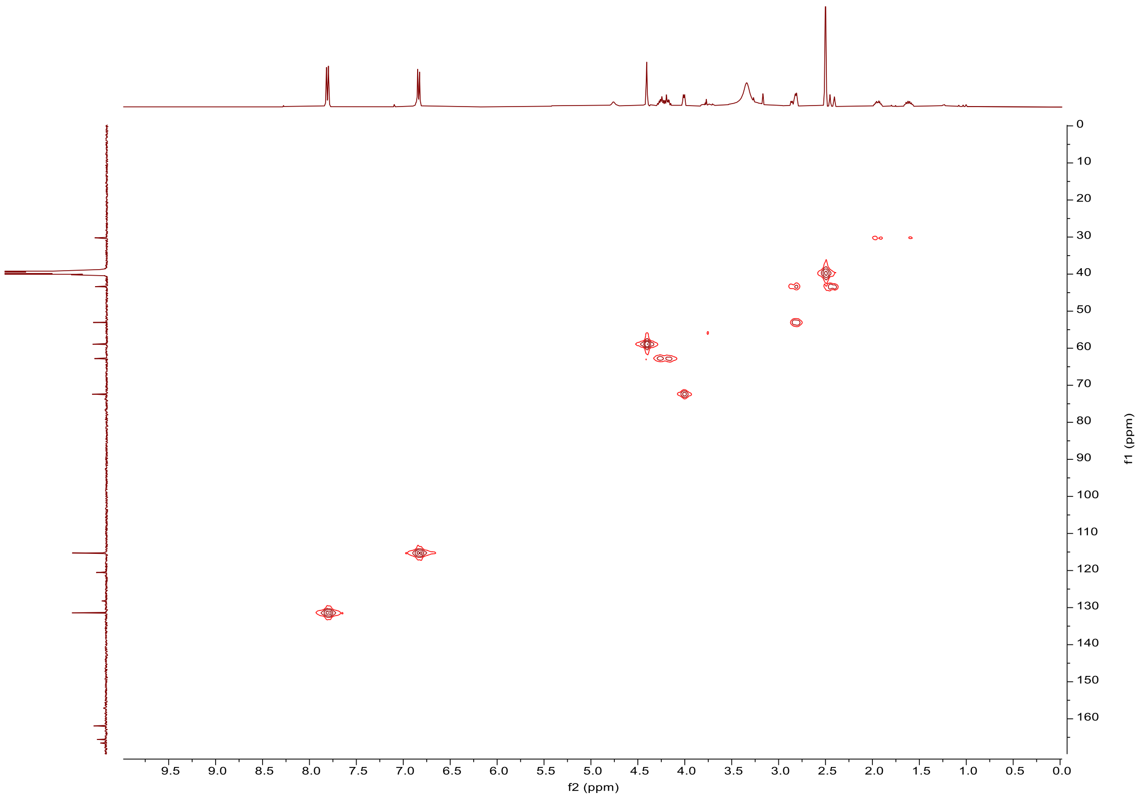

Figure S8. ^{1}H-^{13}C HMQC spectrum of compound **1**.

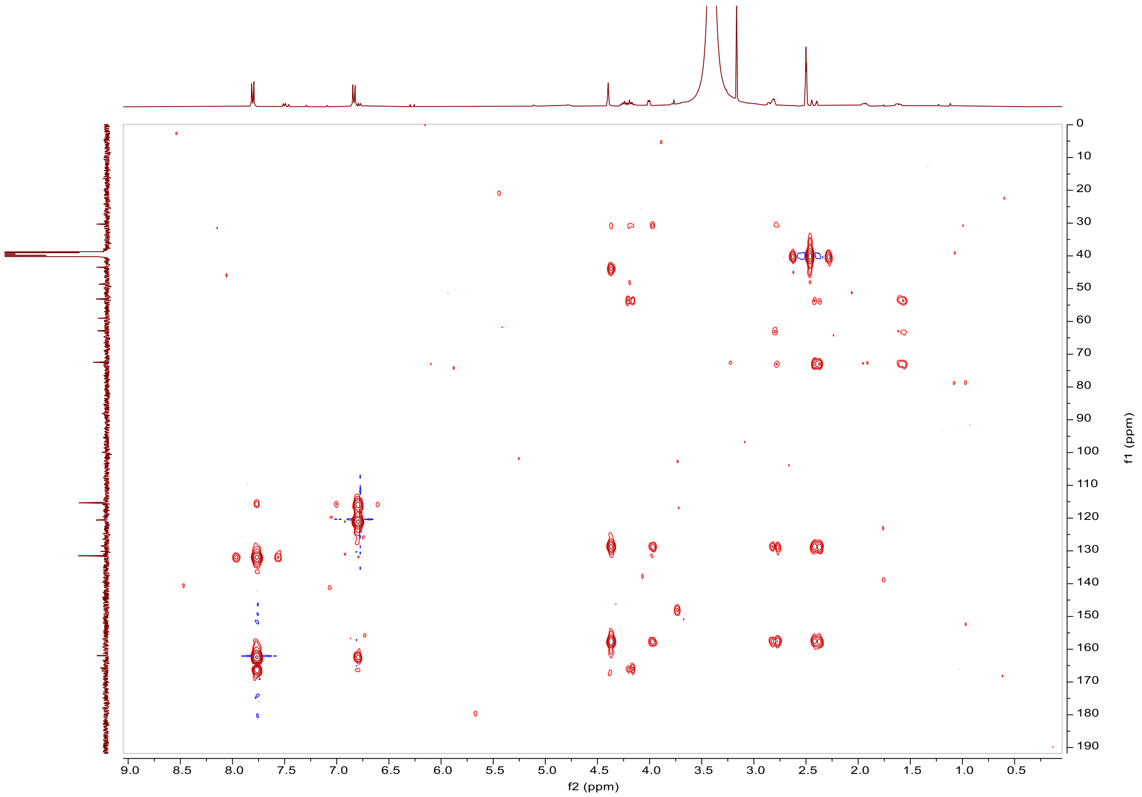

Figure S9. ^{1}H-^{13}C HMBC spectrum of compound **1**.

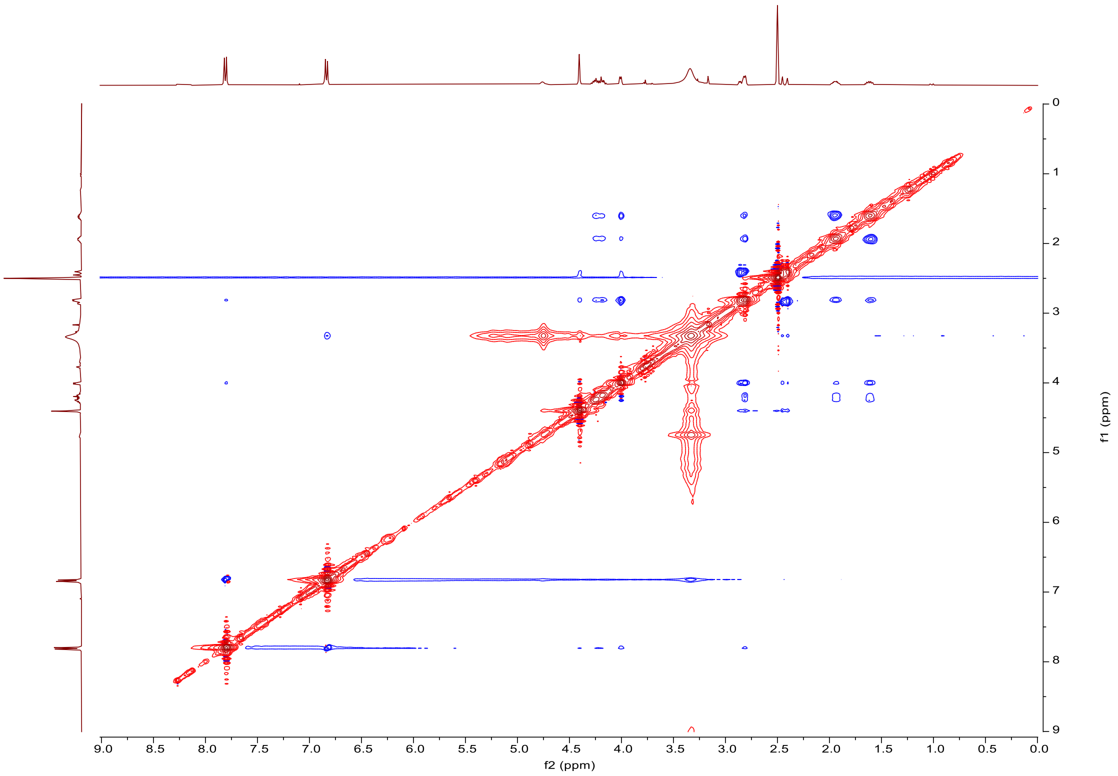

Figure S10. ^{1}H-^{1}H NOESY spectrum of compound **1**.

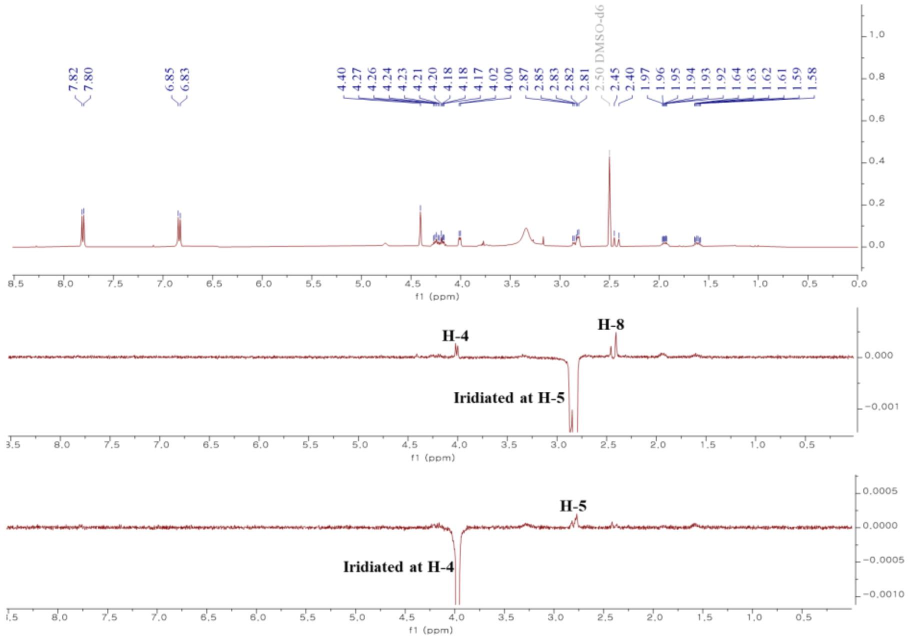

Figure S11. Selective 1D NOE spectrum of compound **1**.

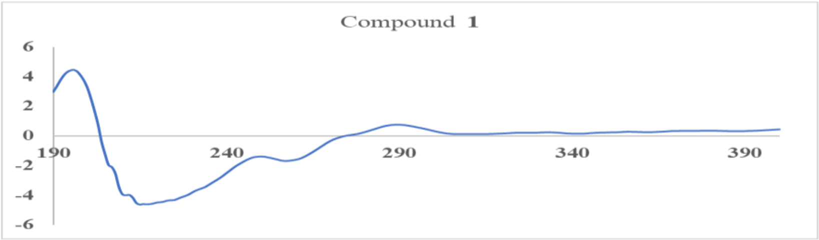

Figure S12. CD spectrum of compound **1**.

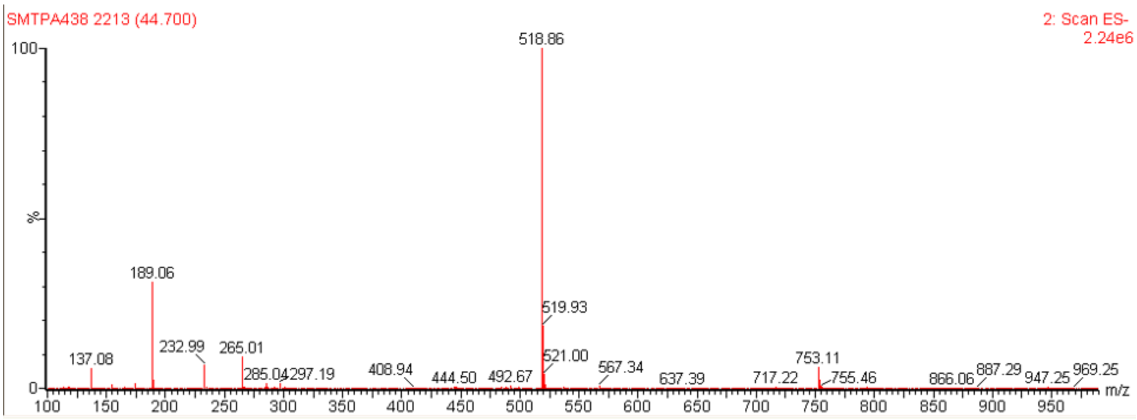

Figure S13. Low mass spectrum of MTPA diester product.

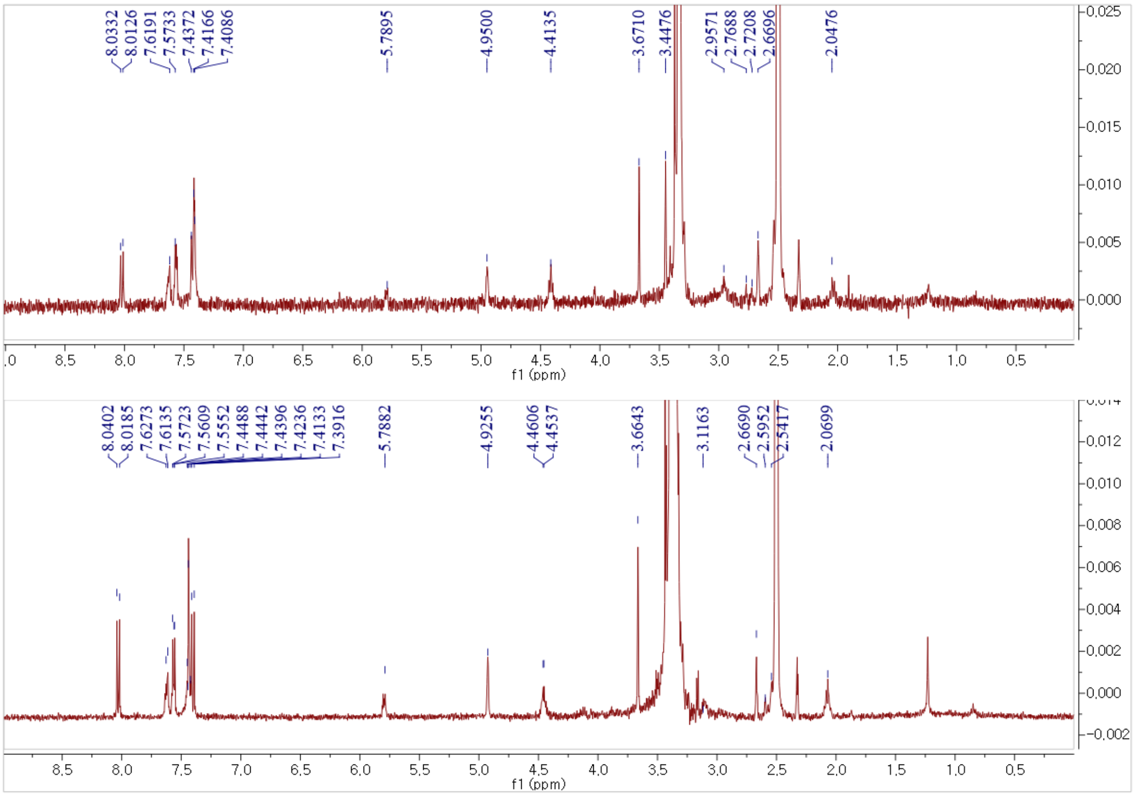

Figure S14. ^{1}H NMR spectra of **1a** (*S*-MTPA) and **1b** (*R*-MTPA) products.

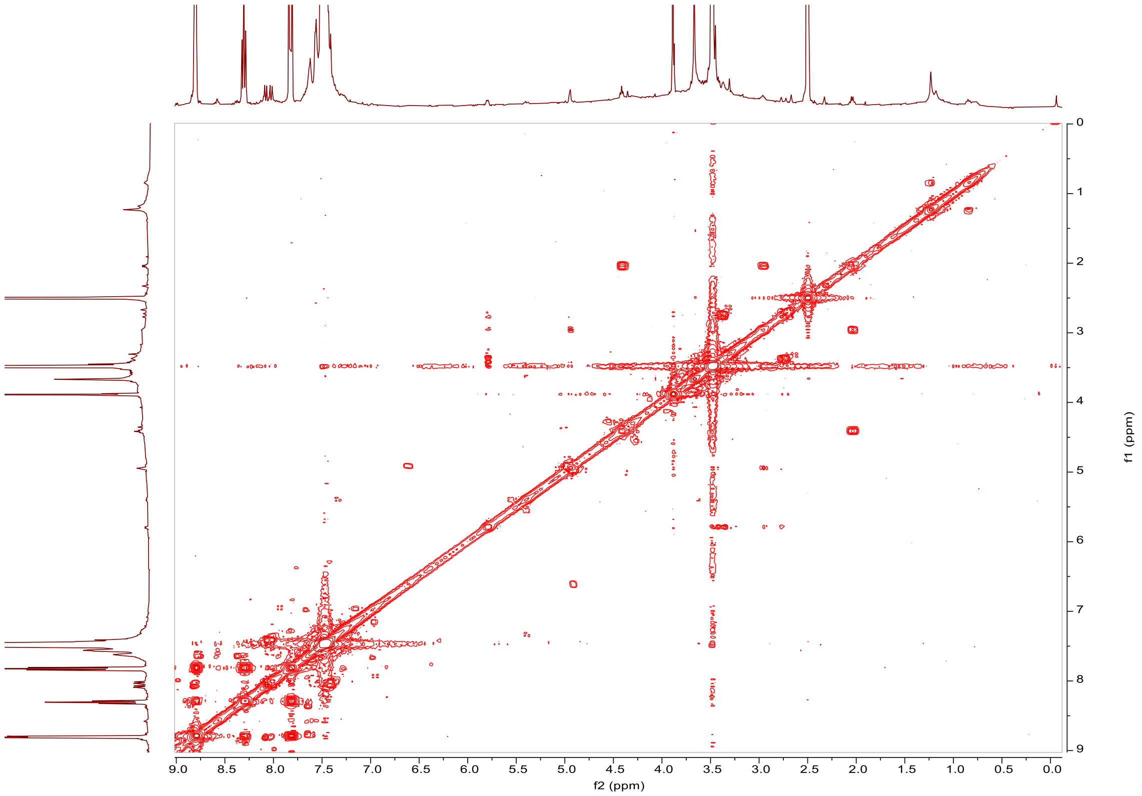

Figure S15. ^1H-^1H COSY spectrum of compound **1a**.

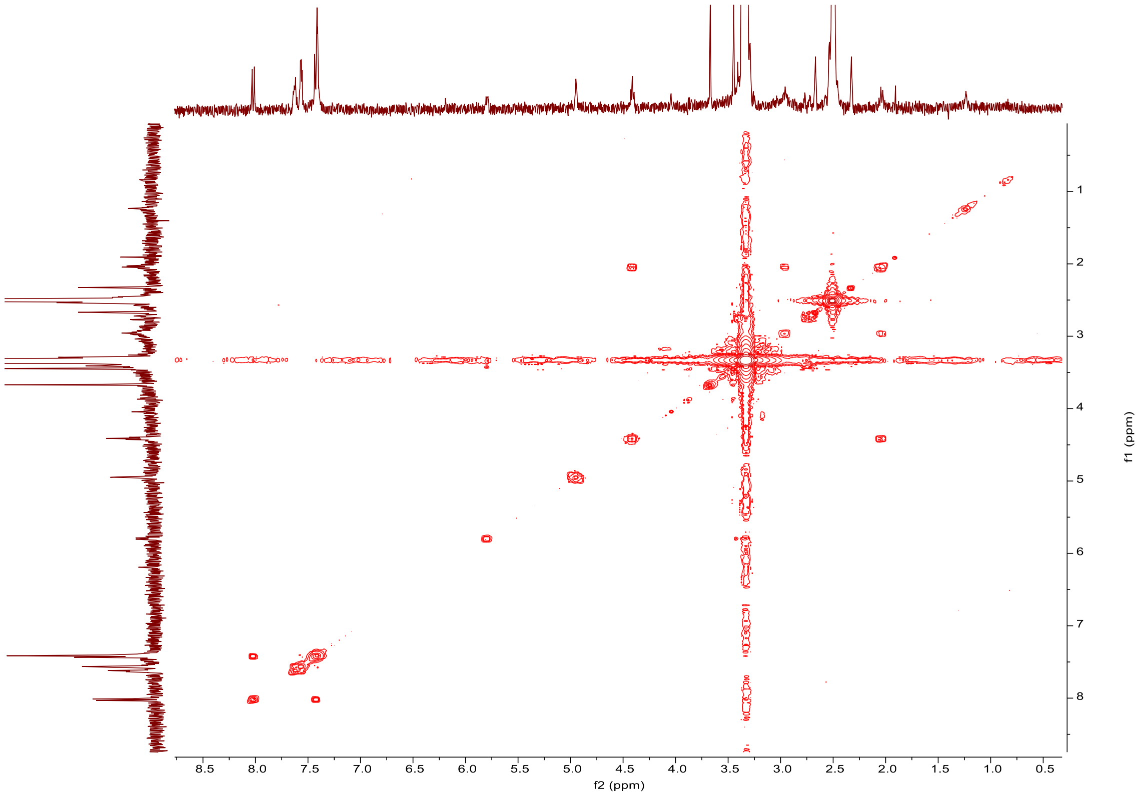

Figure S16. ^1H-^1H COSY spectrum of compound **1b**.
